# Bilateral One-half Spica Cast After Open Reduction and Pelvic Osteotomy in the Developmental Dislocation of the Hip

**DOI:** 10.5812/ircmj.13366

**Published:** 2013-12-05

**Authors:** Hadi Makhmalbaf, Amir Reza Kachooei, Seyed Mahdi Mazloumi, Mohamad Ebrahimzadeh, Farzad Omidi-Kashani, Parham Seyf, Hengameh Ebrahimi, Nastoor Bekhradianpoor, Reza Shiravani Bakhtiari

**Affiliations:** 1 Orthopedic and Trauma Research Center, Mashhad University of Medical Sciences, Mashhad, IR Iran; 2 Department of Educational Nursing, Faculty of Medicine, Mashhad University of Medical Sciences, Mashhad, IR Iran

**Keywords:** Open Reduction, Osteotomy, Bilateral one-half Spica Cast, Dislocations, Development

## Abstract

**Background:**

The prevalence of DDH ranges from 1 per1000 to 3.4 per 100 live- births. One- and-one-half hip spica cast is the conventional technique of immobilization after the open reduction with or without osteotomy.

**Objectives:**

In this study, we evaluated the preference of bilateral one-half spica cast over the one-and-one-half spica cast.

**Materials and Methods:**

Eight hips in five patients with developmental dislocation of the hip have undergone open reduction. Salter osteotomy, Pemberton osteotomy and femoral shortening were done on two, two and six hips, respectively. Bilateral one-half spica cast with trochanteric molding was applied for all of the hips.

**Results:**

The patients’ age ranged from 1.5 to 7 years old. The only complications consisted of two osteonecrosis of the head and one superficial infection. No dislocation, graft displacement, nonunion and device failure was occurred.

**Conclusions:**

Bilateral one-half spica cast is sufficient after the open reduction with or without osteotomy in DDH patients and we can substitute the bilateral one-half spica cast above the knee cast for the conventional one and one-half spica cast.

## 1. Background

Developmental dysplasia of the hip is the common status in the neonates and infants. The word “congenital dislocation of the hip” was replaced by “developmental dislocation of the hip” to comprise the children who are born with a normal hip but dislocates after a while (1). The prevalence of DDH ranges from 1 per 1000 to 3.4 per 100 live- births (2). Higher prevalence was reported after screening by both physical and sonographic examinations (3). In a study on 11868 neonates done by pediatrics, Ortholani sign showed the positive result, from mild click to apparent dislocation, in one per 50 neonates (4). In another study done in Britain, they reported the DDH prevalence to be 7.7% if it is screened by sonography, 8.1% if only neonates with risk factor undergo screening by sonography and 2.1% if only physical examination is used for screening (5). In Sweden, the prevalence is reported to be 1.7 per 1000 live- births (6). The immediate goal in the treatment of a dislocated hip is to obtain concentric reduction as soon as practical (4). Open reduction is indicated if closed reduction is unsuccessful or incomplete (5).

After the open reduction and capsulorraphy with or without osteotomies such as Salter or Pemberton osteotomies, immobilization in a hip spica cast may take at least six weeks for capsular healing and bone union (6).The conventional spica cast which has been already emphasized in reference books and also is practical in most centers, is a one-and-a-half spica cast (1-1/2 spica cast) (7).

## 2. Objectives

In this study, we evaluated the preference of bilateral one-half spica cast over one-and-one-half spica cast in our patients. To the best of our knowledge, this is the first study which introduced bilateral one-half spica cast after surgical treatment of DDH.

## 3. Materials and Methods

Eight hips in five patients with developmental dislocation of the hip have undergone surgery from September 2011 to October 2012 in Ghaem University Hospital, Mashhad, Iran. The patients’ age ranges from 1.5 and 7 years old. None of them had a teratologic dislocation. Three patients had bilateral dislocation. None of them experienced any previous surgery. All of the hips were referred with a complete dislocation. The dislocation was diagnosed by plain x-ray.

### 3.1. Operative and Post-operative Technique

All of the hips underwent open reduction with or without pelvic osteotomy. We used Bikini incision for open reduction and osteotomy. Complete excision of pulvinar in the acetabulum is very important for femoral head to be sited completely and also safe in the acetabular cavity. We tried no manipulation on the labrum and chondero-osseous border of the acetabulum. Some of the hips needed shortening osteotomy; but no derotation or varus correction was done during shortening osteotomy and the osteotomy site was internally fixed by a 4-hole 3.5 DCP. The pelvic osteotomy was either Salter or Pemberton. We used pin fixation with two k-wires after Salter osteotomy. No fixation was used after Pemberton osteotomy. We applied bone graft from either iliac crest or the shortening segment of the femur. At the end, tight capsulorraphy was done. One of the most important parts was that an assistant tried to keep the operated limb in a reduced and safe position until spica cast has been applied. This position has approximately 45 degrees of flexion, 30 degrees of abduction and 10-15 degrees of internal rotation. We applied 1/2-1/2 spica cast. We followed the patients with serial radiography after six and 12 weeks, six and nine months and one year, incessantly.

### 3.2. Spica Cast Application

**Figure 1.**
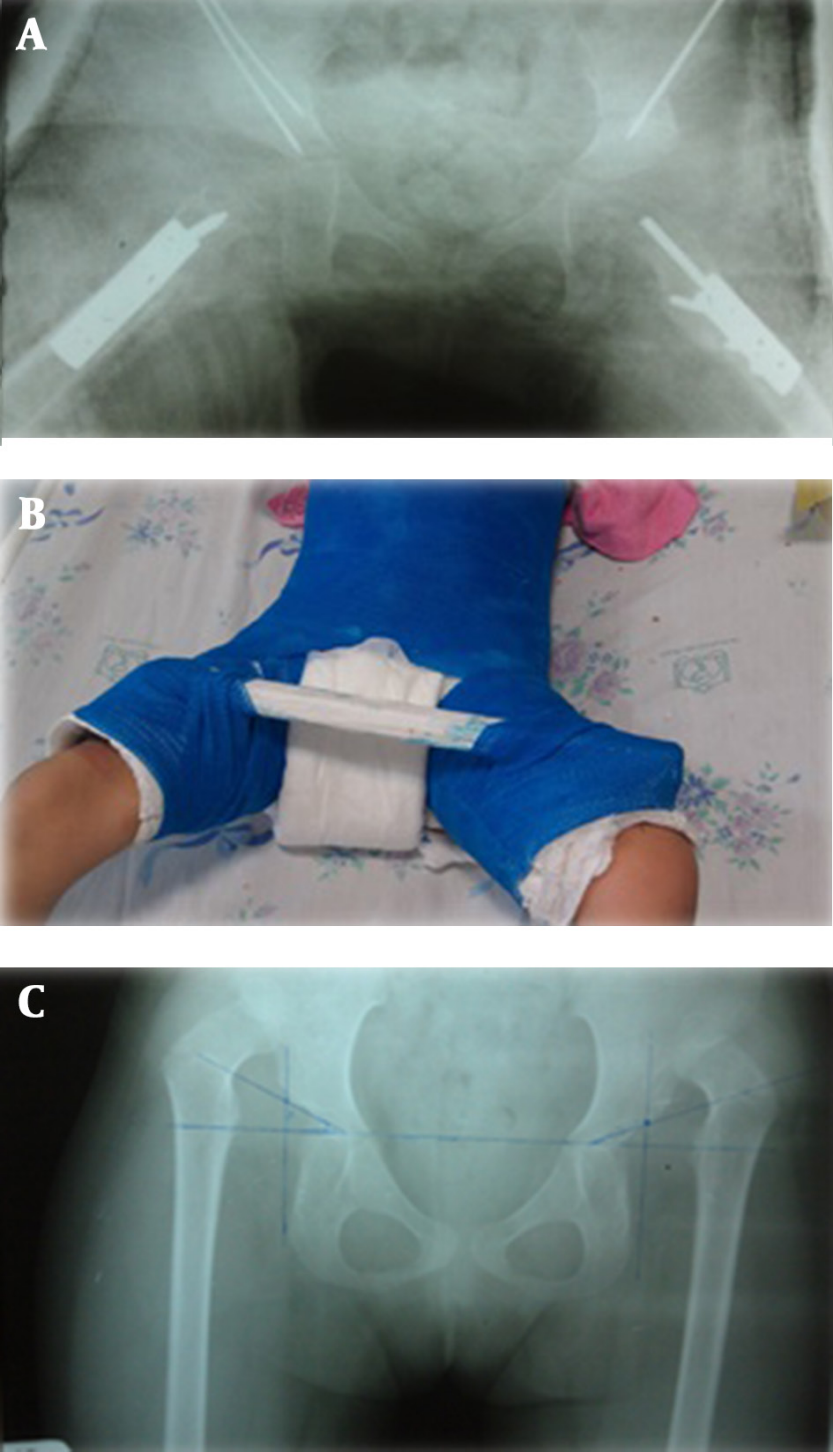
Plain X-ray of a 6.5 Year Old Girl Referred With Bilateral DDH. She Undergone Open Reduction and Salter Osteotomy Bilaterally. Bilateral one-half Spica Cast Was Applied After Both Hips Surgeries

At the end of all surgeries, we applied a spica cast. Our modification technique of casting was applying a 1/2-1/2 spica cast instead of the conventional 1-1/2 spica cast. We applied the cast on both sides from beneath the nipple line to just above the knees. For casting, after applying a 4-inch webril underneath, we used three plaster splints on each side: back to front; from the back of the trunk to the medial side of the femur, front to back; from the front of the abdomen to the lateral side of the femur making a cross with the previous splint and straight from lateral side of the femur up to the chest wall. Then, we applied one layer of a 4-inch plaster cast on the splints and completed the casting with a layer of 4-inch scotchcast on the top surface. Two most important point’s needs to be considered during casting are that an expert assistant or surgeon must keep the operated limb in the safe and reduced position and meanwhile trochanteric molding should be done on the operated hip which is the keystone of the casting. We continued the first spica cast for six weeks. We changed the cast in the operation room for examining the hip and removing the sutures under anesthesia. In unilateral patients, we continued casting for another six weeks , then, the abduction brace was used . In bilateral patients, we went on the operation of the other side after six weeks from the first operation and the casting was continued for another six weeks for both sides. In the third 6-week period, the cast was applied only as a single limb spica (1/2 spica cast) and meanwhile abduction brace was applied at nights (Figure 1 A-C). 

## 4. Results

We operated eight hips in five patients. Three patients had bilateral dislocation. Demographic data are listed in Table 1. 

**Table 1.** Data of the eight Hips in 5 Patients That Undergone Open Reduction With or Without Pelvic Osteotomy. 1/2- 1/2 Hip Spica Cast was Applied for all of the Eight Hips

hips	Gender	Age, y	Involved Side	Salter Osteotomy	Open Reduction	Pemberton Osteotomy	Shortening Femoral Osteotomy	Capsulorraphy	ROM Before Operation
1	Male	7	Left	-	√	-	√	√	↓Ab
2	Female	6.5	Left	√	√	-	√	√	↓Ab
3	Female	6.5	Right	√	√	-	√	√	↓Ab
4	Male	3	Left	√	√	-	√	√	↓Ab
5	Female	1.5	Right	-	√	√	-	√	↓Ab
6	Female	1.5	Left	-	√	√	-	√	↓Ab
7	Female	5	Right	-	√	-	√	√	↓Ab
8	Female	5	Left	-	√	-	√	√	↓Ab

1/2-1/2 hip spica cast was applied for all of the eight hips. According to the postoperative physical examination and radiography, none of the eight hips dislocated in the follow- up period. Union has occurred in all of the osteotomy sites in pelvic and femur. Complications are listed in Table 2.

**Table 2.** Complications in our 8 Operated Hips After Open Reduction and Spica Casting

Complications	Number of Hips
Dislocation	0
Graft displacement	0
Nonunion	0
Femoral head osteonecrosis	2
Device failure	0
Deep infection	0
Superficial infection	1

## 5. Discussion

Bilateral one-half spica cast is sufficient after the open reduction with or without osteotomy in DDH patients and we can substitute the bilateral above the knee cast for the conventional one and one-half spica cast. To the best of our knowledge, this is the first study which introduces the bilateral one-half spica cast after the surgical treatment of DDH. One and one-half spica casting has been already in practice in many centers and it is emphasized on as a postoperative protocol in reference books (2, 7). We did not find any difference, with regard to obtained results, between short and long leg casts; therefore, the advantages of a short cast are obvious. It is lighter and it makes the child’s carriage simpler. Also, the child can sit in different places. The child can move the limb from the knee to avoid joint stiffness and also he or she can feel free in short cast rather than long cast. Besides, the complications at the time of cast removal such as heat injury would disappear from beneath the knee. The other advantage is that by movement of knee and the tension applied on the hamstring, adductors and quadriceps muscle, the head moves and circles inside the acetabulum making it deeper by this pressure.

On the other hand, the technique of casting can contribute a lot in the success of casting (2). Trochanteric molding is the most important part of casting which keeps the femoral head sited deep in the acetabulum during healing period unless the head migrates gradually out of the acetabulum. Proper surgical technique and peri-casting holding of the limb by the assistant are also important parameters forsuccess of casting both in the short and long leg cast. If we consider every simple point from the beginning of the surgery, it is the head which remains in the acetabulum, not the cast. So, casting is just for immobilizing the hip and there is no difference between the short and long leg cast.
